# Characterization of Date Palm Fiber-Reinforced Different Polypropylene Matrices

**DOI:** 10.3390/polym12030597

**Published:** 2020-03-05

**Authors:** Mohammed Sh. Al-Otaibi, Othman Y. Alothman, Maher M. Alrashed, Arfat Anis, Jesuarockiam Naveen, Mohammad Jawaid

**Affiliations:** 1 National Industrialization Company (Tasnee), Riyadh 11496, Saudi Arabia; moham.alotaibi@tasnee.com; 2 Department of Chemical Engineering, College of Engineering, King Saud University, Riyadh 11421, Saudi Arabia; mhalrashed@ksu.edu.sa; 3 SABIC Polymer Research Center, Department of Chemical Engineering, College of Engineering, King Saud University, Riyadh 11421, Saudi Arabia; aarfat@ksu.edu.sa; 4 Department of Mechanical and Manufacturing Engineering, Faculty of Engineering, Universiti Putra Malaysia, Serdang 43400, Selangor, Malaysia; gandhi.naveen66@gmail.com; 5 Laboratory of Biocomposite Technology, Institute of Tropical Forestry and Forest Products (INTROP), Universiti Putra Malaysia, Serdang 43400, Selangor, Malaysia

**Keywords:** date palm fiber, homopolymer polypropylene, copolymer polypropylene, recycled polypropylene, alkali treatment

## Abstract

In this study, the effect of different polypropylene (PP) matrices (homopolymer (HPP), impact copolymer (ICP), and recycled polypropylene (rPP)) on the mechanical, morphological, and thermal properties of date palm fiber (DPF)-reinforced PP composites was investigated. The DPFs were treated with an alkali solution, and composites were fabricated with different DPF loadings (5, 10, and 15 wt %) and lengths (less than 2 mm and 8–12 mm). It was found that the tensile properties of the DPF/ICP and DPF/rPP composites were similar to those of the DPF/HPP composites. The addition of fiber to the matrix reduced its tensile strength but increased the modulus. The alkali treatment improved the compatibility between the fibers and the matrix by removing hemicellulose and other impurities. Fourier transform infrared spectroscopy confirmed hemicellulose removal. The morphology of the alkali-treated fractured tensile specimen revealed improved adhesion and less fiber pull out. Differential scanning calorimetry revealed that the alkali treatment enhanced the crystallinity index. Thermogravimetric analysis showed that the addition of DPFs into the PP matrix reduced the thermal stability of the composite. However, the thermal stability of the treated fiber-reinforced rPP and ICP composites was similar to that of the DPF/HPP composite. Hence, rPP can be used as an alternative to HPP with DPFs.

## 1. Introduction

The utilization of natural resources for industrial applications will enhance the biodegradability of many daily-life disposable products, besides producing new materials with better mechanical, physical, and thermal properties [1]. Although natural fiber-based polymeric composites have existed since 1900, they did not gain much attention until the 1980s. However, nowadays, natural or green composites are extensively utilized owing to eco-legislation, and the benefits of natural fiber-reinforced composites, such as a high strength, modulus, and strength-to-weight ratio, as well as cost-effectiveness and biodegradability [2].

Date palm fibers (DPFs) are abundant in Arab countries, with approximately two-thirds of the date palm trees in the world existing in Arab lands. The date palm is considered the most important palm species after coconut and oil palms [3]. During cultivation and fruit harvesting, date palm trees produce different residues, such as bunch stalks, leaf stalks, and tree trunks. These huge bio-wastes from the date palm trees may pollute the environment [4]. These agro-wastes can be utilized to produce low-cost sustainable and eco-friendly materials [5]. In particular, these agro-wastes can be potential reinforcements for thermoset or thermoplastic polymers for non-load-bearing applications.

Polypropylene is a thermoplastic polymer produced by the polymerization of propylene monomer in the presence of a catalyst under heat and pressure. Propylene (propene) is an unsaturated hydrocarbon containing only carbon and hydrogen. A homopolymer is a polymer that is formed by the polymerization of a single monomer and contains only one kind of repeating unit throughout the polymeric chain [6]. Homo-polypropylene offers a high strength, stiffness, and strength-to-weight ratio compared with its copolymer. In addition, it possesses good chemical resistance and weldability, which render it applicable in several corrosion-resistant structures. Copolymers, also known as heteropolymers, consist of two or more different monomers. The process of joining two monomers is called copolymerization, and the main purpose of this process is to modify the properties of manufactured plastics to meet specific market needs. Polypropylene impact copolymers (ICPs) are produced by the copolymerization of propylene and ethylene monomers in the presence of a catalyst (phthalate-free Ziegler–Natta catalyst). ICPs are used in thermoforming, injection molding, and blow molding. Plastic recycling is a process used to recover plastic scraps for reprocessing and using them to produce valuable products. Polypropylene recycling is one of the most common plastic recycling processes, which mainly includes sorting, collecting, cleaning, and reprocessing of polypropylene wastes [6].

Abu-Sharkh et al. [7] investigated the effect of adding a compatibilizer on the mechanical performance of DPF/polypropylene composites. Initially, the DPF and the polymer were compounded at 200 °C using an extruder. Subsequently, the composite specimens for mechanical testing were injection molded as per the standard dimensions. The mechanical test results showed that the incorporation of a compatibilizer (Epolene E-43) enhanced the mechanical properties. Alawar et al. [8] investigated the effect of NaOH (concentrations: 0.5%, 1%, 1.5%, 2.5%, and 5%) and hydrochloric acid (0.3, 0.9, and 1.6 N) treatments on the mechanical and thermal performances of DPFs for composite reinforcement. The treatments were carried out for 1 h at 100 °C. The mechanical test results revealed that the fibers treated with 1% NaOH had higher strength and modulus. In addition, the thermal stability improved after the alkali treatment. However, an opposite trend was observed in the case of hydrochloric acid treatment, where no such improvements were observed. Abdal-hay et al. [9] investigated the effects of varying DPF diameter (800–600, 600–400, and 400–200 μm) and alkali treatment on the properties of DPFs and its composites. They reported that the DPFs with smaller diameters exhibited higher mechanical properties compared with the coarse fibers. The alkali-treated fiber and composites exhibited an enhanced load-carrying capacity, and consequently, the treated fibers and their composites exhibited higher strength and modulus compared with those of the untreated fibers and their composites. The Fourier transform infrared (FTIR) analysis confirmed that the carbonyl group (C=O) stretching vibration at ~1728 cm^−1^ originating from hemicellulose disappeared due to the alkali treatment.

Nowadays, due to eco-legislation and environmental pollution, many researchers have focused on reducing the utilization of polymeric materials and products. This goal has motivated the researchers to identify promising natural materials which are eco-friendly and cost effective. The purpose of this research is effective utilization of recycled polypropylene in order to reduce the consumption of homopolymer. Hence, this study focuses on evaluating the effects of different polypropylene (PP) matrices (homopolymer (HPP), impact copolymer (ICP), and recycled polypropylene (rPP)) on the mechanical, thermal, and morphological properties of DPF-reinforced polypropylene composites. Moreover, the properties were improved using alkali treatments. Furthermore, this research has proven that rPP can be used as an alternative to HPP with DPFs.

## 2. Materials and Methods 

### 2.1. Materials

Table 1 shows the polymers used in this research and their properties. A commercial homopolymer polypropylene (HPP; melt flow index (MFI): 12 g/10 min) was supplied by TASNEE (Riyadh, Saudi Arabia) and an impact copolymer polypropylene (ICP; MFI: 70 g/10 min) was supplied by SABIC (Riyadh, Saudi Arabia). The above-mentioned HPP was used as a matrix in the DPF-based polymeric composites. In addition, recycled homopolymer polypropylene (rPP) was used to investigate the feasibility of using recycled plastics with natural resources. Composite samples were prepared with different polymer loadings. Exxelor supplied by ExxonMobil (Machelen, Belgium) was used as a coupling agent (3%) in the composite formulations to enhance the compatibility between the fibers and the PP matrix.

DPFs supplied by the Al-Nojoom Factory (Al-Kharj, Saudi Arabia) were used as a filler in the compounding process to prepare the composite samples. For comparison, DPFs of different sizes were used in this work. The raw fibers were milled into two different sizes: powders of less than 2 mm (P) and fibers of 8–12 mm length (F). The DPFs were treated with a 5% NaOH aqueous solution at room temperature for 24 h. Subsequently, the fibers were cleaned with water to remove excess NaOH and heat treated at 70 °C for 72 h.

### 2.2. Fabrication of Date Palm Fiber/Polypropylene Composites 

Table 2 shows the formulations of different composite samples. The DPF content in the PP/DPF composite was varied as 5, 10, and 15 wt %. The compounding process was performed using a twin-screw extruder (Xplore MC 15 micro-compounder Sittard, The Netherlands). The residence time in the extruder was 1 min for each sample and the screw speed was 100 rpm. Tensile test specimens were prepared using an Xplore micro-injection molding machine.

### 2.3. Characterization

#### 2.3.1. Tensile Test

Tensile testing was carried out as per ASTM D638 with a Zwick/Roell Z010 instrument using dog-bone-shaped composite samples [10]. The crosshead speed was maintained at 5 mm/min.

#### 2.3.2. Differential Scanning Calorimetry (DSC)

The thermal properties of DPF/polypropylene composites were investigated by differential scanning calorimetry (TA Instruments, Model DSC-Q200, Malvern, UK). Approximately 5–7 mg of a powdered DPF/PP composite sample was loaded in an aluminum pan, while an empty aluminum pan was used as a reference. DSC was performed in a nitrogen environment from 50 to 195 °C (10 °C/min). The % crystallinity can be determined using the following relation:

%Crystallinity = (ΔHm − ΔHrc)/(xΔHf),

where ΔHm and ΔHrc are the melting enthalpy and secondary crystallization enthalpy during the DSC heating process, respectively; ΔHf is the melting enthalpy of 100% crystallized PP (ΔHf for HPP, rPP, and ICP is 207 J/g (taken from supplier data sheet)); and x is the content of the PP in composite.

#### 2.3.3. Thermogravimetric Analysis (TGA)

The weight loss and thermal stability of the DPF/PP composites were analyzed using a thermogravimetric analyzer as per ASTM E1131-03. Powdered samples of around 20 mg were kept in an alumina crucible and subjected to pyrolysis in N_2_ atmosphere (50 mL min^−1^). The analysis was performed in the temperature range of 30–900 °C at a heating rate of 10 °C/min.

#### 2.3.4. Morphological Analysis

The microstructure and morphology of the DPF/PP composites were investigated using a scanning electron microscope (FEI Quanta 200) at 20 kV. The fractured samples were sputter-coated with gold to visualize the failure modes effectively.

#### 2.3.5. Attenuated Total Reflectance (ATR)-FTIR Analysis

ATR is a widely used FTIR spectroscopy sampling tool. The change in the functional groups after chemical treatment was investigated by ATR-FTIR analysis. 

## 3. Results

### 3.1. Tensile Properties 

**Figure 1:**
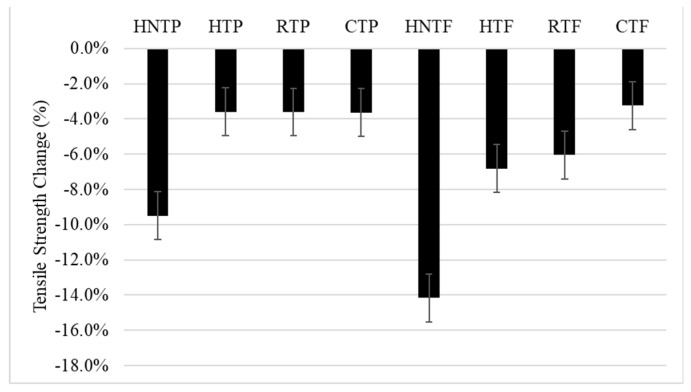
Change in tensile strength.

Figure 1 shows that the tensile strength has declined after DPF (15%) addition in all composite samples. However, the percentage drop in tensile strength varies with respect to the type of matrix. In addition, it has been found that the alkali treatment of the DPFs mitigated the tensile strength reduction. The overall tensile strength reduction is due to the poor interfacial interaction between the hydrophilic DPFs and the hydrophobic polymer matrices [11]. On the other hand, the alkali treatment enhanced the adhesion between the DPFs and polymeric matrices by removing hemicelluloses and other impurities. In addition, it cleared the micro-pores in the fibers [12].

**Figure 2:**
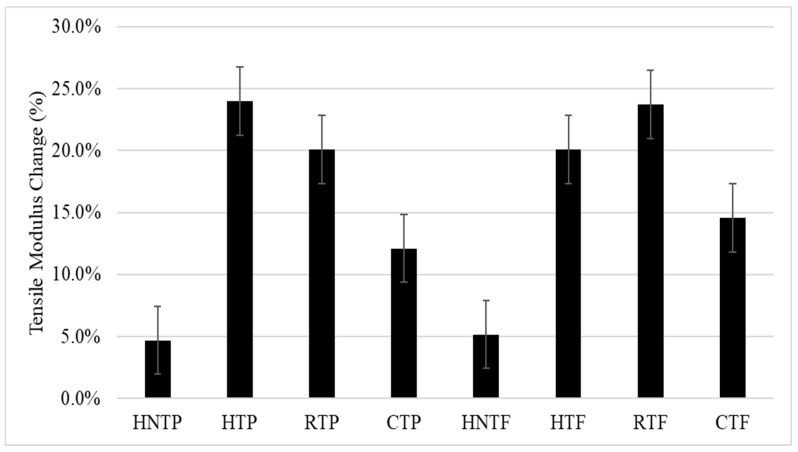
Change in tensile modulus.

Figure 2 presents the tensile moduli of different DPF/PP composites. The tensile moduli of all the composites increased, especially the treated fibers. Both the HTP and HTF composites exhibited higher moduli among the composites. Nonetheless no significant difference was observed between the tensile properties, as shown in Figure 1 and Figure 2, of the treated DPF/rPP and DPF/HPP composites.

#### 3.1.1. Effect of DPF Loading and Treatment on Tensile Properties

**Figure 3:**
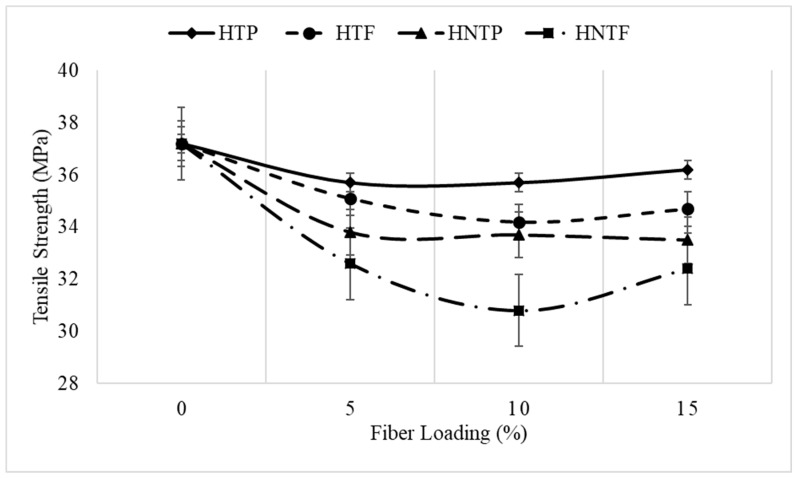
Effect of treatment and fiber loading on tensile strength.

Figure 3 displays the variation in tensile strength with fiber loading. The tensile strength decreased with an increase in fiber content, which is mainly due to the incompatibility or poor interfacial interaction between the hydrophobic polymer and the hydrophilic fiber.

Figure 3 shows the mechanical performances of the PP/raw fiber (untreated) composite and PP/alkali-treated fiber composite as a function of fiber loading. As can be seen, for both the fiber sizes and at all fiber loadings, the treated fiber performed better than the untreated fiber. Alkali treatment enhanced the interfacial adhesion between the fiber and polypropylene matrix [13,14].

**Figure 4:**
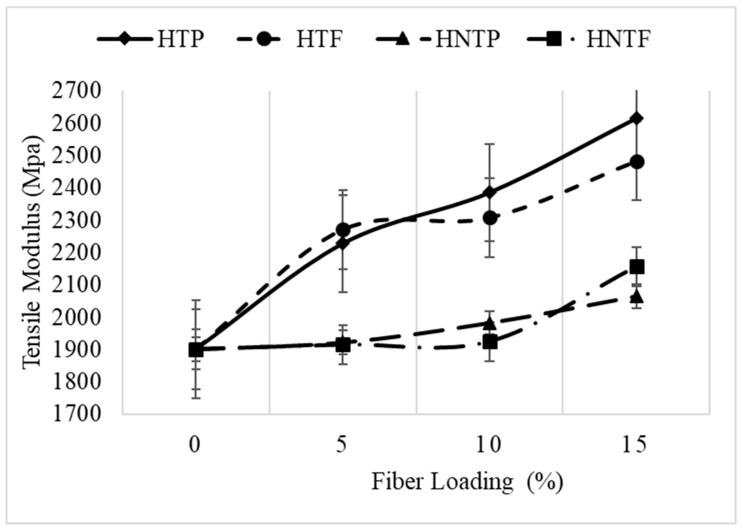
Effect of treatment and fiber loading on tensile modulus.

Figure 4 shows the tensile modulus at various fiber loadings. As can be seen, the tensile modulus increased with an increase in fiber loading from 5% to 15%, which is consistent with the findings of Abu Sharkh et al. [7] who reported an increasing trend up to 30% loading.

#### 3.1.2. Effect of Fiber Loading and Matrix Type on Tensile Properties

**Figure 5:**
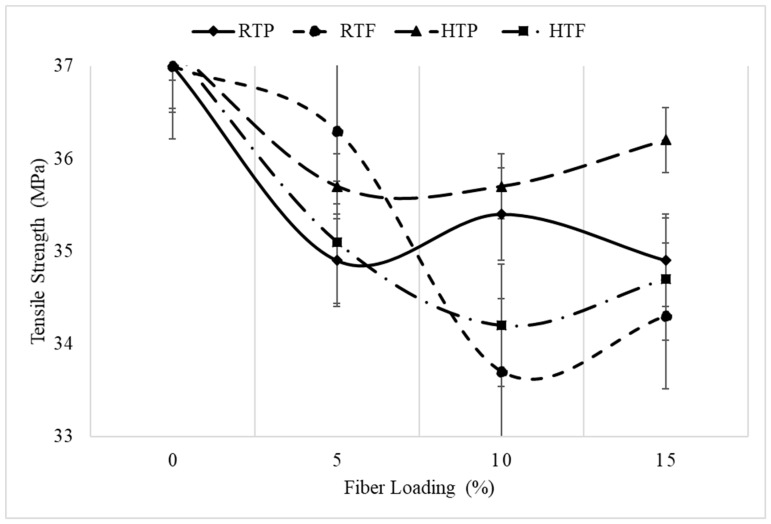
Tensile strengths of virgin and recycled polypropylene (PP) composites.

Figure 5 shows the tensile strength at different fiber loadings and for different matrices (HPP and rPP). As can be seen, the tensile strength decreased with an increase in fiber loading for both the fiber sizes. Moreover, rPP exhibited a similar trend as that of HPP.

**Figure 6:**
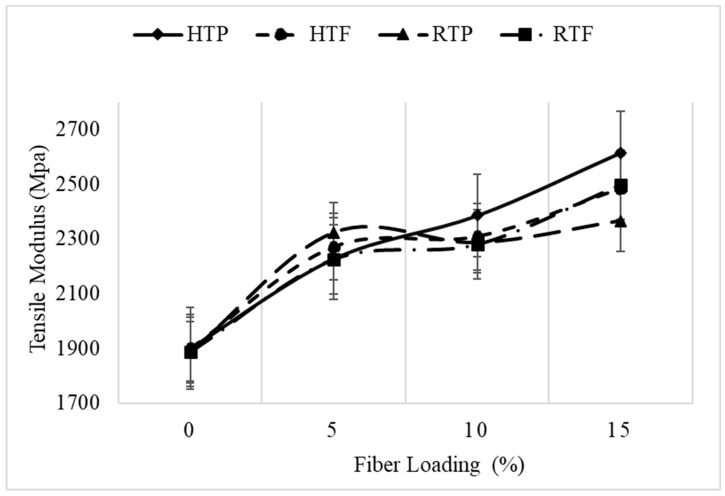
Tensile moduli of virgin and recycled PP composites.

Figure 6 shows the tensile modulus for various fiber loadings. The tensile modulus increased with an increase in fiber content for both the virgin and recycled PP matrices, and both of them exhibit the same trend, which proves that the tensile modulus is unaffected by the recycling process. 

### 3.2. Morphological Analysis

**Figure 7:**
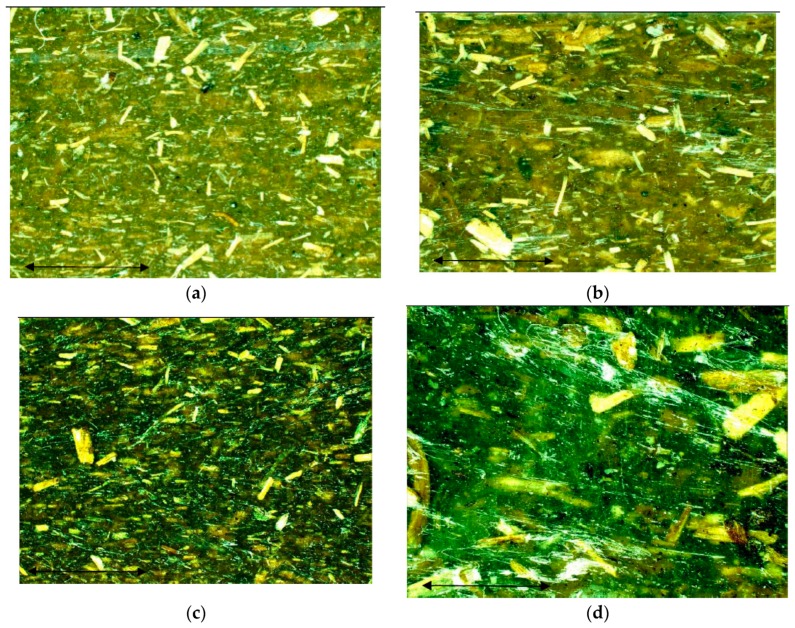
Surface microscopy images of HPP composite with 15% loading of date palm fiber (DPF). (**a**) Untreated powder; (**b**) untreated fiber; (**c**) treated powder; (**d**) treated fiber.

Figure 7 shows that the untreated fibers accumulated on the surface of the composite specimens, which indicates the incompatibility between the fiber and the polymer. The treated fibers, on the other hand, only slightly accumulated on the surface of the composite specimens, which indicates that the alkali treatment enhanced the compatibility between the fiber and the polymer.

**Figure 8:**
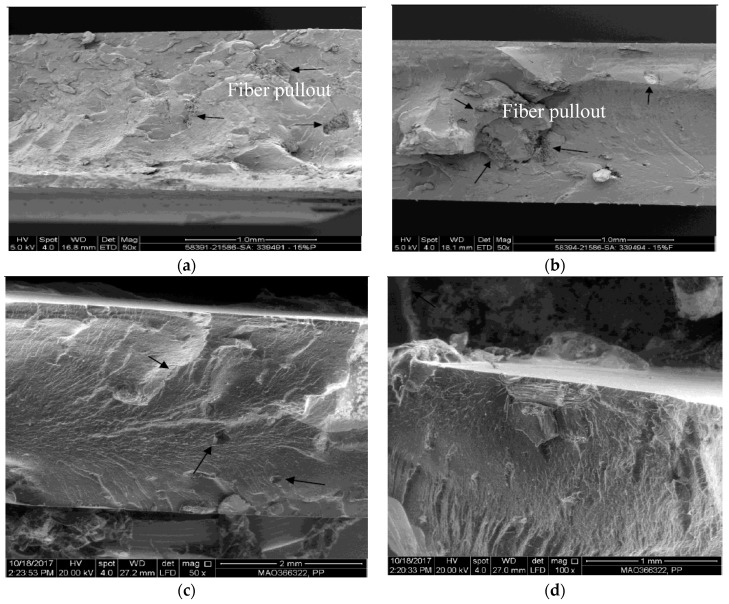
SEM images of fractured surfaces of HPP composite with 15% loading of DPF. (**a**) Untreated powder; (**b**) untreated fiber; (**c**) treated powder; (**d**) treated fiber.

Similarly, the fracture cross-section SEM images of the of the untreated fibers, as shown in Figure 8, show extensive fiber pull out and a rough surface, which explains the reduction in tensile strength due to fiber loading [7]. In addition, irregular fiber shapes in the matrix indicate improper mixing. Proper mixing plays a vital role in improving the adhesion between the fiber and the polymeric matrix [15]. The SEM images of the composites reinforced with the treated fibers exhibit smoother fracture surfaces with minimal fiber pullout compared with the untreated fibers owing to the enhancement in interfacial adhesion [7].

### 3.3. Attenuated Total Reflection (ATR)-FTIR Analysis

**Figure 9:**
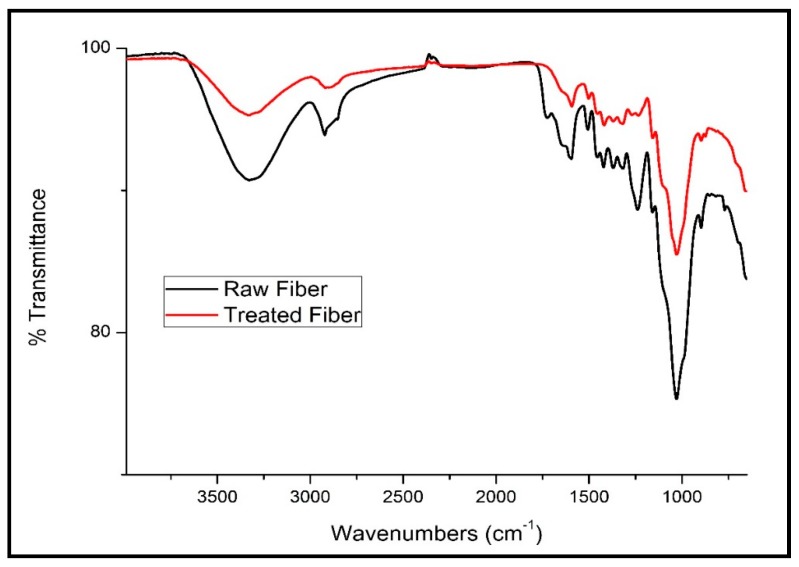
Attenuated total reflectance (ATR)–FTIR spectra of raw and alkali-treated fibers.

ATR analysis is a FTIR spectroscopy sampling technique that can show the differences in the chemical structures of the treated and untreated fibers. Figure 9 displays the ATR-FTIR spectra of the raw and alkali-treated DPFs. Table 3 shows the different bands and their corresponding functional groups. The wide absorption band at 3329 cm^−1^ is due to O–H stretching and can be seen in the spectra of both the treated and untreated fibers with a slight difference. The peak at 2921 cm^−1^ is attributed to the aliphatic C–H stretching vibration, while the peak at 1724 cm^−1^ is associated with the carbonyl group (C=O) stretching vibration. Nonetheless, the spectrum of the treated fibers shows a reduction in the absorption of the carbonyl group due to the removal of hemicellulose after the NaOH treatment. The peak at 1238 cm^−1^ is associated with the bending vibration of the CH_3_ group. These findings are similar to the FTIR spectroscopy results of coir and date palm fibers [16].

### 3.4. Differential Scanning Calorimetry (DSC)

#### 3.4.1. Non-Treated Fiber/PPH Composite 

Table 4 shows that addition of untreated fibers in the homo polypropylene matrix has no significant impact on the crystallinity index. It may be due to the fact that impurities present in the surface of the untreated fiber reduces the contact area with the polymeric matrix [17].

#### 3.4.2. Treated Fiber/PPH Composite

Table 5 shows the crystallinity index of treated fibers/PPH composites. DSC results show that the crystallinity index has been increased significantly after the alkali treatment and this can be explained by the dissolution of hemicellulose, which consequently promoted the breakdown of the fiber into micro fibrils. Due to this phenomenon the effective contact surface has improved. Furthermore, the crystalline cellulosic chains rearrange themselves, thereby enhancing the crystallinity index [18]. In addition to that, fibrillation and disappearance of amorphous hemicellulose improved the aspect ratio of the date palm fiber.

#### 3.4.3. Treated Fiber/Recycled PPH Composite

Table 6 shows the crystallinity index of treated fibers/PPH recycled matrix composites. DSC results show that the crystallinity index has been increased in a way similar to the prime matrix. Thus, there is no significant difference between the recycled and prime matrices’ interactions with the treated fibers.

#### 3.4.4. Treated Fiber/ICP Composite

Table 7 shows the crystallinity index of treated fiber/ICP composites. DSC results show that addition of treated fibers to the ICP matrix has insignificant impact on the crystallinity index. These results can validate the tensile modulus results.

### 3.5. Thermogravimetric Analysis (TGA)

TGA analysis was carried out to investigate the thermal stability of untreated DPF/PPH, treated DPF/PPH, treated DPF/recycled PPH, and treated DPF/ICP composites at different DPF wt % and lengths.

#### 3.5.1. Non-Treated Fiber/PPH Composite

**Figure 10:**
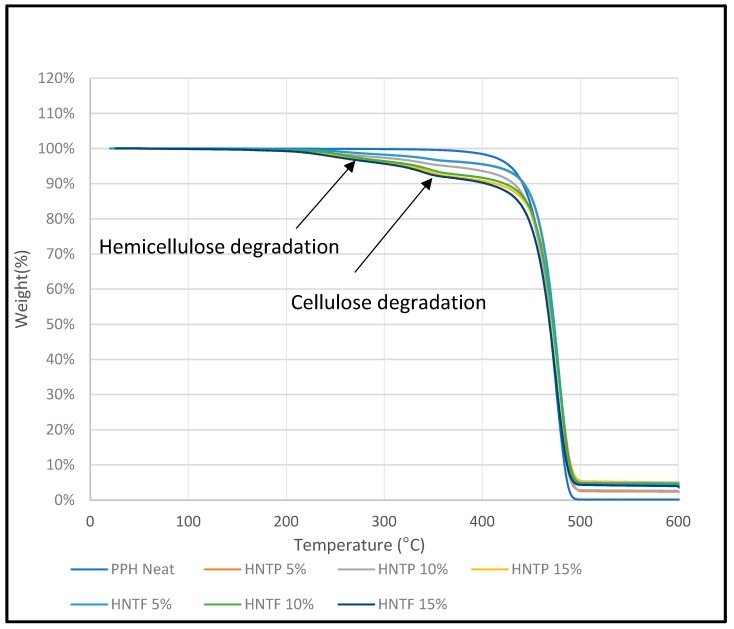
TGA of homo polypropylene-non-treated fiber composites.

From the thermogram shown in Figure 10, it is clear that PPH samples exhibited major degradation shifts (around 400 °C) compared to other composite samples. Addition of fibers in the PPH matrix has declined the thermal stability of the composites. It can be attributed to the degradation of natural chemicals, such as cellulose, hemicellulose, and lignin present in the date palm fiber. The initial degradation around 100–150 °C was associated with the evaporation of water molecules [19]. Then, the natural chemicals, such as hemicellulose and cellulose, were degraded at 290 and 360 °C, respectively [20]. The major degradation occurs in between 350 and 450 °C for all the composite samples with minimal deviations. The polypropylene matrix degraded around 400 °C. The sample at 300 °C has lost 4% of its weight due to the degradation of hemicellulose and other components, which degrade before 300 °C.

#### 3.5.2. Treated Fiber/PPH Composite

**Figure 11:**
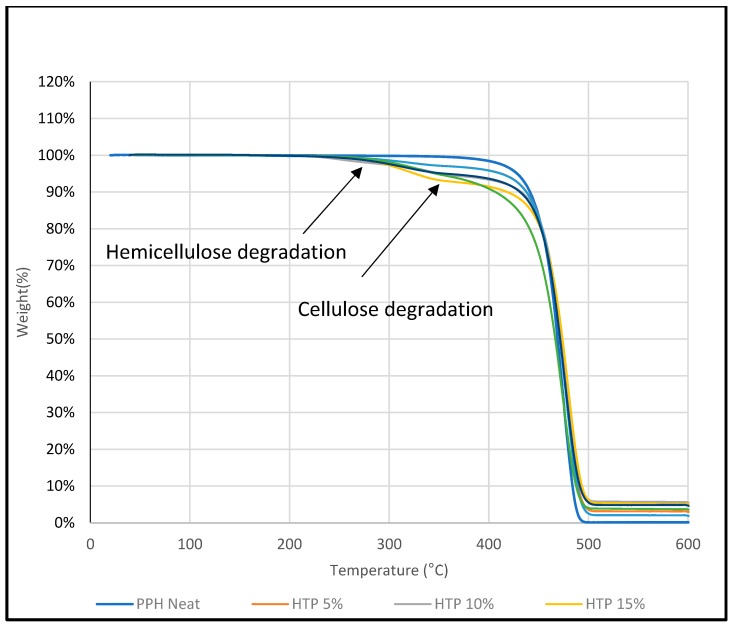
TGA of homo polypropylene-treated fiber composites.

Figure 11 shows that addition of treated fibers into the PPH matrix has decreased the thermal stability of the composite, but it is clear that the treatment mitigated the degradation by hemicellulose removal, which usually degrades at around 290 °C. Furthermore, it is good to know that the reason that makes the composite degradation start before 300 °C is not only the hemicellulose, but also the lignin, which decomposes slower, and over a broader temperature range (200–500 °C) [21] than cellulose and the hemicellulose components. At 300 °C, all the treated fiber-reinforced composite samples exhibited only 2% weight loss. On the other hand, untreated DPF-reinforced homo polypropylene composites showed 4% weight loss at 300 °C. It has corroborated the fact that treated fiber enhanced the thermal stability of the composites.

#### 3.5.3. Treated Fiber/Recycled PPH Composite

**Figure 12:**
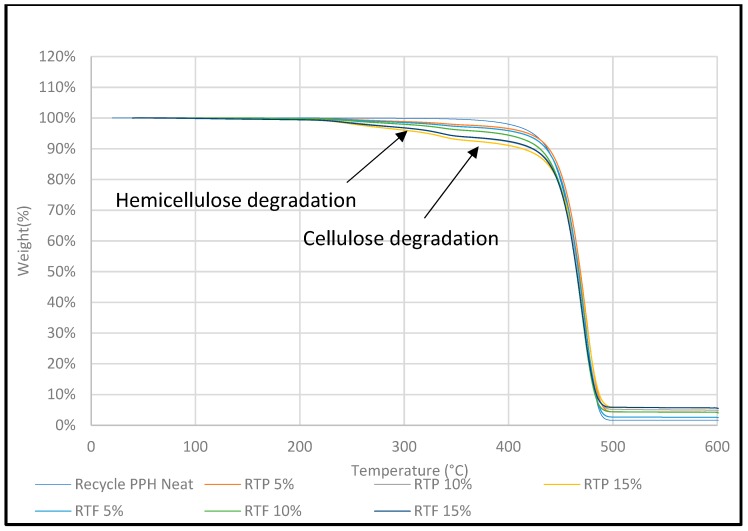
TGA of recycled homo polypropylene-treated fiber composites.

Figure 12 shows that the thermal degradation profile and thermal stability of recycled PPH /DPF composite is almost similar to PPH/DPF composites, as shown in Figure 12. In particular, the major degradation temperature was found at 420–450 °C for both recycled PPH/DPF composites and PPH/DPF composites, irrespective of the wt % of fiber and length. Hence, using recycled PPH with the treated fibers instead of prime matrix did not change the thermal stability of the composite, which proves that recycling did not affect the thermal stability of the homo polypropylene matrix [6]. Thus, recycled PPH can be utilized as an alternative to the prime material with DPF. 

#### 3.5.4. Treated Fiber/ICP Composite

**Figure 13:**
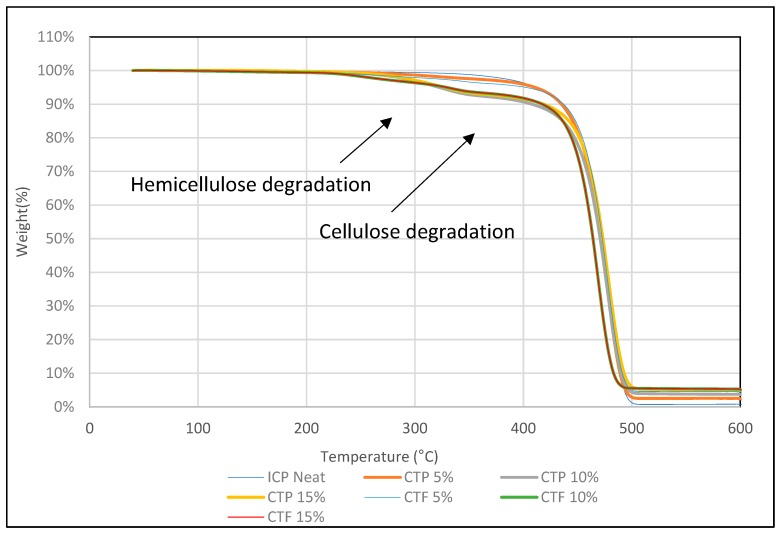
TGA of ICP-treated fiber composites.

Figure 13 shows that neat ICP exhibited higher thermal stability compared to treated fiber-reinforced ICP composites. Nonetheless, the major degradation temperature was observed in between 420 and 450 °C for all the treated DPF/ICP composite samples. Thus, impact copolymer matrix with the treated fibers showed similar thermal stability and degradation curves compared to homo or copolymer with DPF.

#### 3.5.5. Matrices’ Comparison 

**Figure 14:**
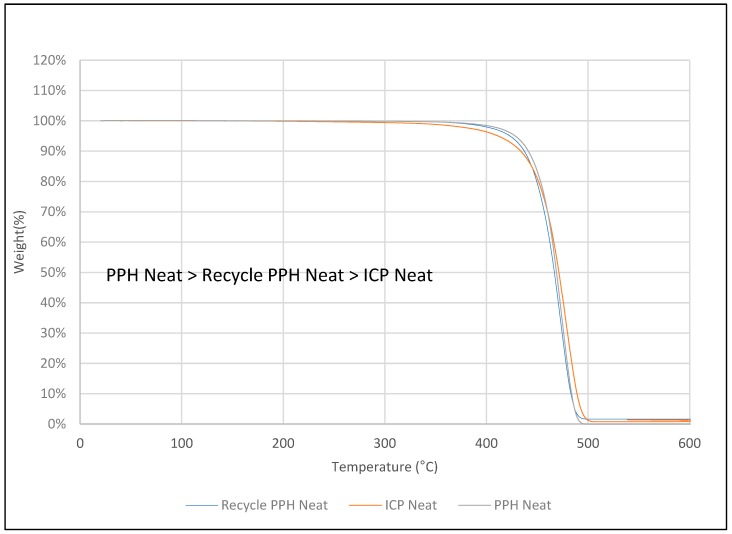
TGA of different PP matrices.

Figure 14 shows the thermogram of different PP matrices. It is clearly understood that PPH has shown the better thermal stability, followed by recycled PPH and ICP. However, no significant difference in degradation temperature was observed. PPH, recycled PPH, and ICP follows a similar degradation profile with slight deviations. The major degradation temperature is in between 420 and 450 °C for all the polypropylene samples. The final degradation temperature was found beyond 450 °C. However, beyond 500 °C, all the polypropylene samples were degraded completely.

Derivative thermogravimetric (DTG) analysis was carried out to investigate the thermal stability of various DPF/PP composites with different DPF sizes and loadings.

**Figure 15:**
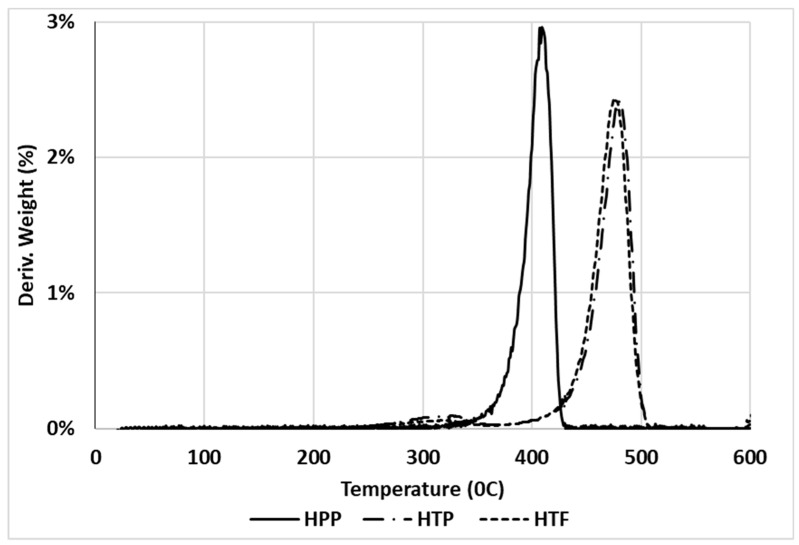
Derivative thermogravimetric (DTG) curves of treated DPF/HPP composites.

**Figure 16:**
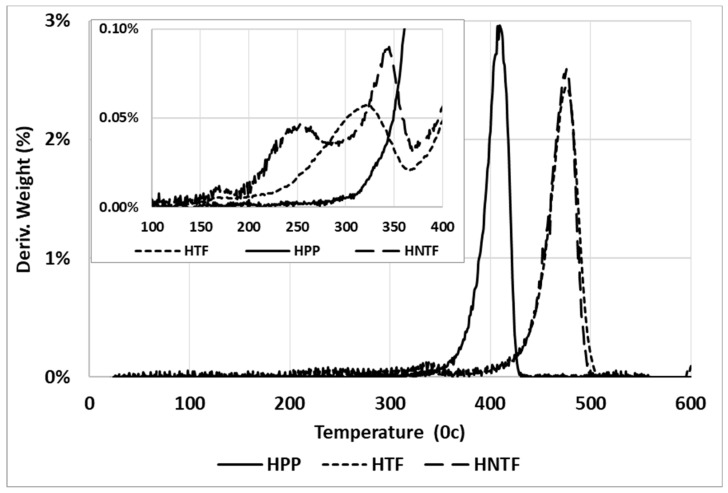
DTG curves of treated and untreated DPF/HPP composites.

Figure 15 and Figure 16 show that the HPP degradation peak (around 400 °C) shifted toward higher temperatures after the addition of DPFs. In general, DPF/polymer composites exhibit three stages of degradation. The initial degradation around 100–150 °C is associated with the evaporation of water molecules [19]. This stage was not observed, which indicates enough drying. However, the other two degradation stages were observed. First, the natural components, such as hemicellulose and cellulose, degraded at 290 and 360 °C, respectively [20]. Then, a major degradation occurred between 350 and 450 °C for all the composite samples with minimal deviations. The polypropylene matrix degraded around 400 °C. Similar results were observed from the DTG curves of the composites for both the fiber sizes. 

It is evident that the treatment mitigated degradation through hemicellulose removal, which usually degrades at around 290 °C. In addition, the composite degradation started before 300 °C, which is because of not only hemicellulose, but also lignin that decomposes slowly and over a broader temperature range (200–500 °C) [21] compared with the cellulose and hemicellulose components. At around 300 °C, the treated fiber-reinforced composite samples exhibited only 2% weight loss, while the untreated DPF-reinforced composites showed 4% weight loss; this indicates that the treated fibers enhanced the thermal stability of the composites. Similar results were observed for the rPP and ICP composites.

Hence, the use of rPP with the treated fibers instead of virgin HPP did not change the thermal stability of the composites, which proves that recycling does not affect the thermal stability of the HPP matrix. Thus, rPP can be used as an alternative to the virgin polymer with DPFs.

## 4. Conclusions

The effect of different DPF sizes on various types of polypropylene matrices was investigated. Based on the results, the following conclusions were drawn:

- The alkali treatment of DPFs improved the tensile properties of the homo-polypropylene, co-polypropylene, and rPP composites. An increase in fiber loading decreased the tensile strength and increased the modulus.
- The microscopy images showed that the untreated fibers accumulated on the surface of the composite specimens, which indicates the incompatibility between the fiber and the polymer matrix. However, the alkali treatment of fiber improved the adhesion between the fibers and the matrices.
- The ATR-FTIR analysis results indicated that after the chemical treatment, the functional groups present in the DPFs strongly interacted with the polypropylene matrix.
- The DSC results further proved that the alkali treatment improved the crystallinity index of the DPF/PP composites.
- The TGA results showed that the thermal stabilities of the treated fiber-reinforced rPP and ICP composites are comparable to that of the DPF/homo-polypropylene composite.

Thus, recycled polypropylene can be used as an alternative to homo-polypropylene with DPFs.

## Figures and Tables

**Table 1:** Polymers used with melt index, density, and co-monomer types.

Polymer	Type	Melt Index (g/10 min @ 230 °C, 2.16 kg)	Density (kg/m^3^)
TASNEE PP	Homopolymer (HPP)	12	900
Industrial Recycle of TASNEE PP	Homopolymer (rPP)	12	900
SABIC PP	Copolymer (ICP)	70	905

**Table 2:** Formulations of composite samples.

Sample Designation	Fiber Loading %		
HNTP	5	10	15
HNTF	5	10	15
HTP	5	10	15
HTF	5	10	15
RTP	5	10	15
RTF	5	10	15
CTP	5	10	15
CTF	5	10	15

Matrix: H = homo polypropylene, R = recycled polypropylene, C = impact copolymer. Date palm fiber size: P = powder, F = fiber. Treatment: T = treated, NT = untreated. Example: HNTF = homo-PP with untreated date palm fiber.

**Table 3:** Infrared transmittance bands (cm^−1^) of raw and alkali-treated DPFs.

Bond Type	Raw Fiber	Treated 5% Alkaline
-OH stretching	3329	3331
C = H vibration	2921	2918
C = O stretching	1724	-
C = C stretching	1595	1592
C-H bending	1238	1234
C-C stretching	1029	1028

**Table 4:** Crystallinity index of non-treated fibers/PPH composites.

Sample	Tm (°C)	Tc (°C)	ΔH_m_	Crystallinity %
PPH Neat	163.04	123.33	90.36	43.7%
HNTP 5%	163.37	122.39	84.13	42.8%
HNTP 10%	163.36	122.19	80.51	43.2%
HNTP 15%	161.71	122.61	70.91	40.3%
HNTF 5%	163.07	122.62	82.44	41.9%
HNTF 10%	162.53	122.51	79.83	42.9%
HNTF 15%	161.59	122.46	76.09	43.2%

**Table 5:** Crystallinity index of treated fibers/PPH composites.

Sample	Tm (°C)	Tc (°C)	ΔH_m_	Crystallinity %
PPH Neat	163.04	123.33	90.36	43.7%
HTP 5%	162.67	123.89	89.47	45.5%
HTP 10%	163.4	123.63	81.46	43.7%
HTP 15%	162.11	123.74	76.54	43.5%
HTF 5%	162.67	123.22	97.1	49.4%
HTF 10%	162.25	122.96	89.09	47.8%
HTF 15%	163.16	123.66	80.54	45.8%

**Table 6:** Crystallinity index of treated fibers/PPH recycled matrix composites.

Sample	Tm (°C)	Tc (°C)	ΔH_m_	Crystallinity %
Recycled PPH Neat	162.78	123.51	83.78	40.5%
RTP 5%	162.43	123.8	72.79	37.0%
RTP 10%	162.98	123.73	77.98	41.9%
RTP 15%	162	123.85	80.23	45.6%
RTF 5%	162.32	123.64	79.93	40.6%
RTF 10%	161.57	123.73	81.47	43.7%
RTF 15%	162.36	124.04	81.43	46.3%

**Table 7:** Crystallinity index of treated fibers/ICP matrix composites.

Sample	Tm (°C)	Tc (°C)	ΔH_m_	Crystallinity %
ICP Neat	161.98	123.12	83.95	40.6%
CTP 5%	161.89	123.41	74.81	38.0%
CTP 10%	161.9	124.09	75.08	40.3%
CTP 15%	162.47	124.19	72.97	41.5%
CTF 5%	161.26	122.91	77.53	39.4%
CTF 10%	162.11	122.78	73.08	39.2%
CTF 15%	161.78	122.98	71.39	40.6%
